# Descriptive Epidemiology of Travel and Non‐Travel Related SARS‐CoV‐2 Gamma (P.1/501Y.V3) Variant Cases in England, 2021

**DOI:** 10.1111/irv.13308

**Published:** 2024-05-12

**Authors:** Nurin Abdul Aziz, Katherine Twohig, Mary Sinnathamby, Asad Zaidi, Shirin Aliabadi, Natalie Groves, Sophie Nash, Simon Thelwall, Gavin Dabrera

**Affiliations:** ^1^ COVID‐19 Vaccines and Epidemiology Division, Clinical and Public Health Group United Kingdom Health Security Agency London UK; ^2^ Public Health England COVID‐19 National Epidemiology Cell London UK; ^3^ TARZET Division, Clinical and Public Health Group United Kingdom Health Security Agency London UK

The Gamma (P.1/501Y.V3) variant of severe acute respiratory syndrome coronavirus 2 (SARS‐CoV‐2) was first detected through whole‐genome sequencing (WGS) in Japan in early January 2021 among a group of travellers arriving from Brazil [1]. This was later reported in more than 50 countries, including England.

Early evidence from Brazil suggested that Gamma was associated with higher transmissibility and propensity for re‐infection [2] as well as a possible increased likelihood of hospitalisation [3]. Considering the potential increased risks, further investigation into the epidemiology of the variant in other settings was necessary. Herein, we describe the epidemiology of the Gamma variant in England to 31 August 2021.

Individuals infected with the Gamma variant were identified through genotyping by polymerase chain reaction (PCR) and WGS from the national COVID‐19 Genomics UK Consortium (COG‐UK) sequencing initiative. These data were linked to demographic information held in UK Health Security Agency's Second Generation Surveillance System (SGSS) [4].

Travel exposure was derived from Passenger Location Forms (PLFs), required for entry to the United Kingdom, and routine NHS Test and Trace (T&T) surveys. For cases with no known travel from these sources, information came from additional follow up by UKHSA's Health Protection Teams (HPTs).

Imported cases were defined as confirmed Gamma cases with international travel into England within 14 days before the earlier of symptom onset or specimen date. Secondary cases were those who had contact with a PCR‐confirmed SARS‐CoV‐2 case who had travelled within 14 days of symptom onset or specimen date. Sporadic Gamma cases had no history of travel nor contact with a SARS‐CoV‐2 infected traveller. Only T&T and HPT surveys could confirm secondary and sporadic cases. Cases without confirmation of travel status were excluded from the analysis.

Descriptive analysis was stratified by travel exposure, with imported and secondary cases grouped as travel‐related, and examined by demographic factors including, age, sex, region and place of residence. Residence at the time of testing was identified by test location information or unique property registration number (UPRN); the latter was also used to derive data on residential clustering to assess potential onwards transmission based on initial travel status [5].

Between May and October 2021, the United Kingdom assigned Red, Amber and Green (RAG) categories to countries with perceived risk for importation of SARS‐CoV‐2 variants. Returning travellers from Red‐listed countries required hotel quarantine in Managed Quarantine Facilities (MQF) and those returning from Amber‐listed countries had at‐home quarantine. In this analysis, travel‐related cases were assigned a RAG category based on the highest rating of the countries involved in their journey at the time of travel [6]. Where RAG assignments were unavailable, an assignment of Red was given to those who travelled from a country on the initial travel ban list of countries associated with the Gamma variant [7].

Between 12 February and 31 August 2021, there were 251 confirmed Gamma cases detected in England. Excluding cases with no confirmation of travel status (*n* = 75, 29.9%), 88 (35.1%) were imported, 14 (5.6%) were secondary travel cases, and 74 (29.5%) were sporadic. Early Gamma cases were mostly imported or secondary cases (Figure 1). There were no reported deaths among confirmed Gamma cases in England during the study period.

**FIGURE 1:**
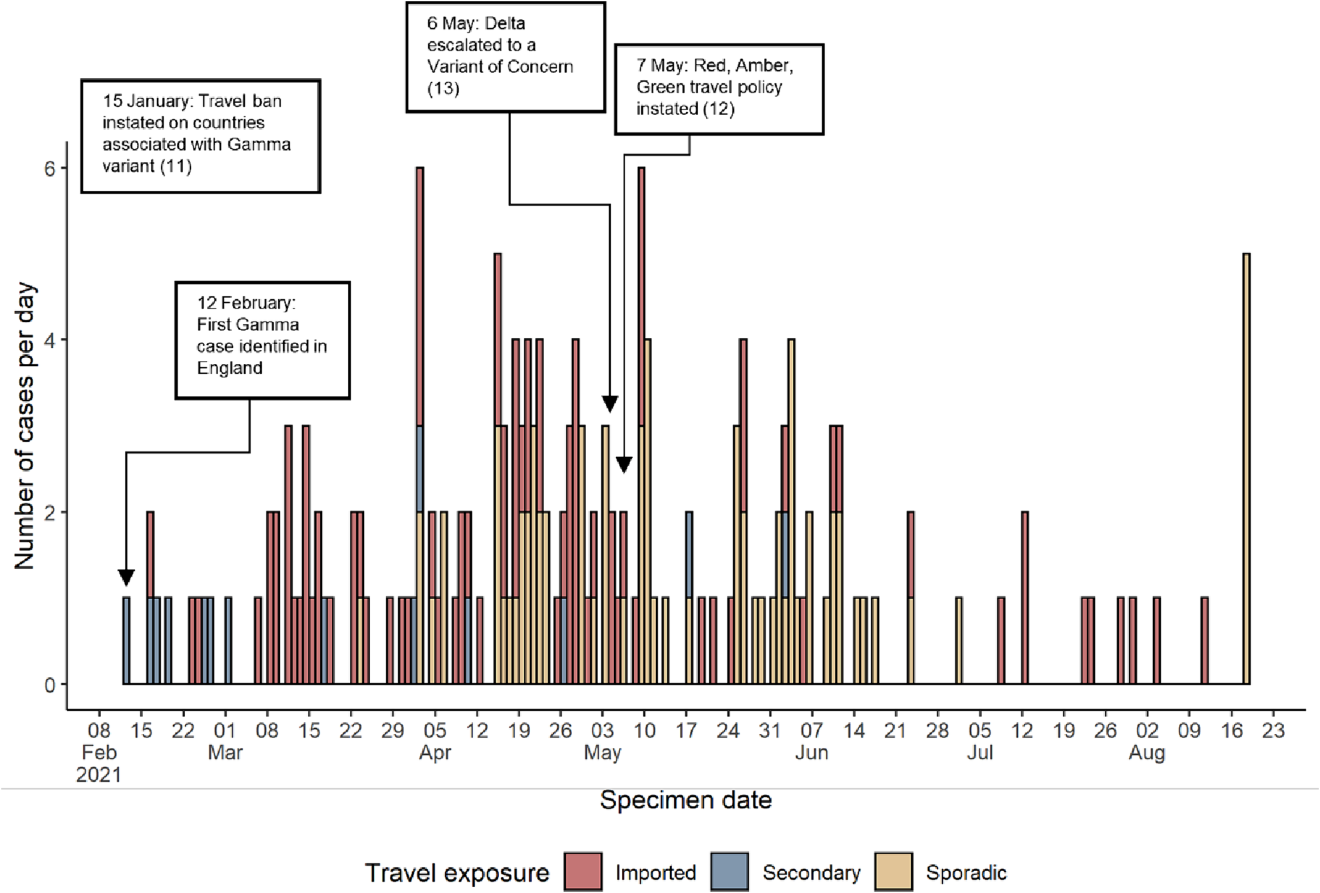
SARS‐CoV‐2 Gamma (P.1) cases with known travel status, England, 12/02/2021–31/08/21.

Those aged between 20 and 29 years had the highest number of travel‐related (*n* = 33, 32.4%) and sporadic cases (*n* = 19, 25.7%). Approximately half of both travel‐related and sporadic cases were from the London region (Table S1).

Contrary to the expected areas of highest risk for importation, the majority of travel‐related cases were associated with Amber countries as the highest RAG rating for their journeys (*n* = 52, 51.0%) (Table S1). However, 40.2% (*n* = 41) of travellers had visited Red countries, including the most frequently reported country of travel, Brazil (*n* = 27, 21.4%), which had been on the Red list since May 2021. Mexico, an Amber country upgraded to Red on 8 August 2021, had 15 returning travellers (11.9%), followed by Amber countries USA (*n* = 14, 11.9%) and France (*n* = 12, 11.1%). These travel‐related cases include those detected within MQFs as part of the travel restrictions.

A larger proportion of sporadic cases were part of a residential cluster (*n* = 31, 41.9%) compared to travel‐related cases (*n* = 28, 27.5%). Cluster size that included sporadic cases ranged from 2 to 6.

This analysis shows that earlier cases of the Gamma variant in England were predominantly travel‐associated, transitioning to a larger proportion of cases likely obtained through community transmission. However, Gamma did not have widespread expansion, despite initial concerns of increased virulence and transmissibility. This may be due to the rapid dominance of Delta, which was observed to have surpassed Gamma in other parts of the world where the two variants co‐occurred in greater numbers, such as Brazil and Mexico [8, 9]. At its peak, Gamma comprised of 2.8% of all cases in England with a variant designation, whereas in the same time period Delta was at 80.4% (Figure S1).

However, the proportion of travel‐associated compared with sporadic cases may have been biased by targeted sequencing of new arrivals and their contacts as well as routine testing of travellers at the time of study [10]. Community‐transmitted cases have potentially lower ascertainment, as they are subject to randomised population‐level sample‐picking processes. However, during the peak of Gamma incidence between March and June, the proportion of positive tests that were sequenced was above 50%, which should mitigate some of the risk for lower detection.

In October 2021, the RAG policy removed Amber and Green categories and only required quarantine for travellers from Red‐list countries [6]. The analysis indicates the policy requiring quarantine in MQFs for Red‐list travellers and at‐home quarantine for Amber‐list travellers, implemented from 7 May 2021, may have contributed to preventing onward transmission as suggested by the small numbers of secondary cases from the time of the policy being introduced. However, within the limitations of the available data, we are unable to definitively conclude which factors most contributed to the decline of Gamma detections in England. Further research is needed to explore the association between quarantine policies and secondary transmission of SARS‐CoV‐2 in other settings.

While larger scale surveillance of travellers has ceased, a similar approach could also be considered when new variants emerge, including using existing surveillance systems such as surveys to monitor the travel status of variant cases.

## Author Contributions

**Nurin Abdul Aziz:** conceptualization, formal analysis, methodology, writing–original draft, writing–review and editing. **Katherine Twohig:** data curation, methodology, writing–original draft, writing–review and editing. **Mary Sinnathamby:** methodology, writing–original draft, writing–review and editing. **Asad Zaidi:** data curation, methodology, writing–original draft, writing–review and editing. **Shirin Aliabadi:** data curation, methodology, writing–review and editing. **Natalie Groves:** data curation, formal analysis, methodology, resources, software, writing–review and editing. **Sophie Nash:** methodology, writing–original draft, writing–review and editing. **Simon Thelwall:** methodology, supervision, writing–original draft, writing–review and editing. **Gavin Dabrera:** conceptualization, methodology, supervision, writing–review and editing.

## Conflicts of Interest

The author declares no conflicts of interest.

### Peer Review

The peer review history for this article is available at https://www.webofscience.com/api/gateway/wos/peer‐review/10.1111/irv.13308.

## Supporting information


**Table S1** Demographic description of SARS‐CoV‐2 Gamma (P.1) cases in England. Data up to 31 August 2021.
**Figure S1.** Proportions and counts of SARS‐CoV‐2 cases by variant designation over time in England from 12 February 2021 to 30 August 2021.
