# Supplementary material for: Descriptive Epidemiology of Travel and Non‐Travel Related SARS‐CoV‐2 Gamma (P.1/501Y.V3) Variant Cases in England, 2021
Source: Influenza Other Respir Viruses. 2024 May 12;18(5):e13308. doi: 10.1111/irv.13308 (PMC11089272; doi:10.1111/irv.13308)
Supplement: Supplementary file 1 — Table S1 Demographic description of SARS‐CoV‐2 Gamma (P.1) cases in England. Data up to 31 August 2021. Figure S1. Proportions and counts of SARS‐CoV‐2 cases by variant designation over time in England from 12 February 2021 to 30 August 2021. [file IRV-18-e13308-s001.docx]

**Supplementary Documents**

**Supplementary Table 1.** Demographic description of SARS-CoV-2 Gamma (P.1) cases in England. Data up to 31 August 2021.

|  | **Imported/Secondary (n = 102)** | | **Sporadic (n = 74)** | |
| --- | --- | --- | --- | --- |
| **Age** | n | % | n | % |
| <10 | 4 | 3.9 | 6 | 8.1 |
| 10-19 | 8 | 7.8 | 15 | 20.3 |
| 20-29 | 33 | 32.4 | 19 | 25.7 |
| 30-39 | 31 | 30.4 | 15 | 20.3 |
| 40-49 | 12 | 11.8 | 9 | 12.2 |
| 50-59 | 9 | 8.8 | 8 | 10.8 |
| 60-69 | 2 | 2.0 | 2 | 2.7 |
| 70-79 | 3 | 2.9 | 6 | 8.1 |
| 80+ | 4 | 3.9 | 15 | 20.3 |
| **Sex** |  |  |  |  |
| Female | 44 | 43.1 | 37 | 50.0 |
| Male | 58 | 56.9 | 36 | 48.6 |
| Unknown | 0 | 0.0 | 1 | 1.4 |
| **Region*** |  |  |  |  |
| London | 57 | 55.9 | 39 | 52.7 |
| Midlands and East of England | 15 | 14.7 | 14 | 18.9 |
| North of England | 4 | 3.9 | 10 | 13.5 |
| South of England | 21 | 20.6 | 9 | 12.2 |
| Unknown | 5 | 4.9 | 2 | 2.7 |
| **Property classification** | | | | |
| Residential dwelling (inc. houses, flats) | 56 | 54.9 | 68 | 91.9 |
| Managed quarantine facilities | 25 | 24.5 | 0 | 0.0 |
| Other property classifications | 4 | 3.9 | 2 | 2.7 |
| Undetermined | 17 | 16.7 | 4 | 5.4 |
| **RAG assignment**** | | | | |
| Red | 41 | 40.2 | - | - |
| Amber | 52 | 51.0 | - | - |
| Green | 1 | 1.0 |  |  |
| Unknown | 8 | 7.8 | - | - |

* The nine regions of England were aggregated to form four larger regions to suppress small numbers and facilitate comparison: North of England (North West, North East England), Midlands and East of England (East Midlands, West Midlands, East of England), South of England (South West, South East England), and the London region.

** Red Amber Green (RAG) assignments based on the country with the most restrictive travel rules in a traveller’s journey (i.e. Red > Amber > Green); this includes journeys of imported variant cases and journeys of travellers in contact with secondary cases.


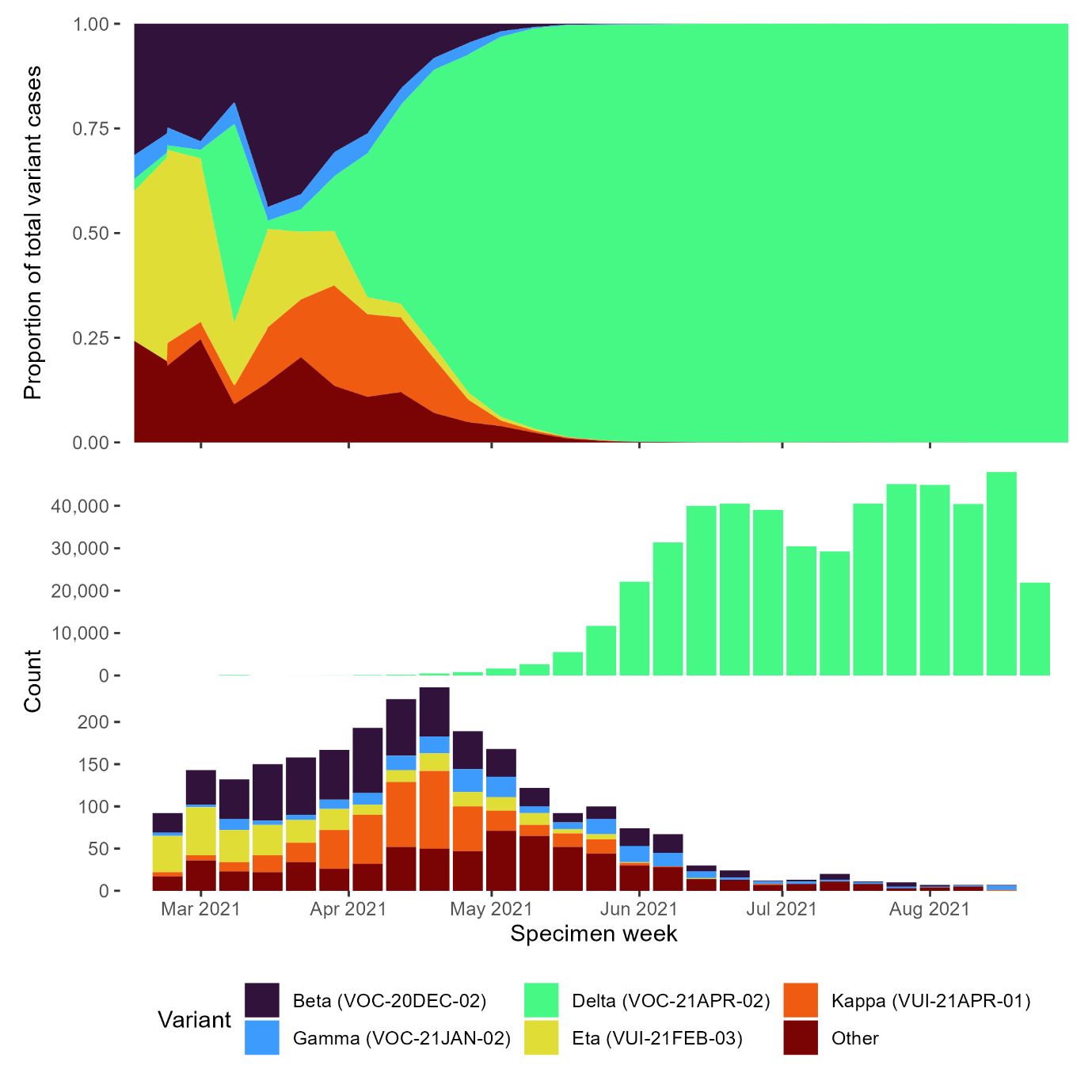


**Supplementary Figure 1.** Proportions and counts of SARS-CoV-2 cases by variant designation over time in England from 12 February 2021 to 30 August 2021.
